# ADORA2A-AS1 Restricts Hepatocellular Carcinoma Progression *via* Binding HuR and Repressing FSCN1/AKT Axis

**DOI:** 10.3389/fonc.2021.754835

**Published:** 2021-10-18

**Authors:** Jian Pu, Ya Zhang, Anmin Wang, Zebang Qin, Chenyi Zhuo, Wenchuan Li, Zuoming Xu, Qianli Tang, Jianchu Wang, Huamei Wei

**Affiliations:** ^1^ Department of Hepatobiliary Surgery, Affiliated Hospital of Youjiang Medical University for Nationalities, Baise, China; ^2^ Graduate College of Youjiang Medical University for Nationalities, Baise, China; ^3^ Department of Pathology, Affiliated Hospital of Youjiang Medical University for Nationalities, Baise, China

**Keywords:** hepatocellular carcinoma, long noncoding RNA, progression, HuR, Fascin, AKT pathway

## Abstract

**Background:**

Hepatocellular carcinoma (HCC) is one of the most aggressive malignancies. Increasing evidence revealed that long noncoding RNAs (lncRNAs) were frequently involved in various malignancies. Here, we explored the clinical significances, roles, and mechanisms of lncRNA ADORA2A antisense RNA 1 (ADORA2A-AS1) in HCC.

**Methods:**

The clinical significances of ADORA2A-AS1 in HCC were analyzed using RNA sequencing (RNA-seq) data from The Cancer Genome Atlas (TCGA) project. The expressions of ADORA2A-AS1, Fascin Actin-Bundling Protein 1 (FSCN1), Matrix Metallopeptidase 2 (MMP2), and Baculoviral IAP Repeat Containing 7 (BIRC7) in HCC tissues and cells were measured by qRT-PCR. Cell Counting Kit-8 (CCK-8), 5-ethynyl-2’-deoxyuridine (EdU), caspase-3 activity assay, transwell migration and invasion assays, and xenograft growth and metastasis experiments were performed to evaluate the roles of ADORA2A-AS1 in HCC. RNA pull-down, RNA immunoprecipitation, qRT-PCR, Western blot, and RNA stability assay were performed to elucidate the mechanisms of ADORA2A-AS1 in HCC.

**Results:**

ADORA2A-AS1 was identified as an HCC-related lncRNA, whose low expression was correlated with advanced stage and poor outcome in HCC. Gain- and loss-of functional experiments demonstrated that ADORA2A-AS1 inhibited HCC cell proliferation, induced cell apoptosis, repressed cell migration and invasion, and repressed xenograft growth and metastasis *in vivo*. Mechanistically, ADORA2A-AS1 competitively bound HuR (Hu Antigen R), repressed the binding of HuR to FSCN1 transcript, decreased FSCN1 transcript stability, and downregulated FSCN1 expression. The expression of FSCN1 was negatively correlated with ADORA2A-AS1 in HCC tissues. Through downregulating FSCN1, ADORA2A-AS1 repressed AKT pathway activation. Functional rescue assays showed that blocking of FSCN1/AKT axis abrogated the roles of ADORA2A-AS1 in HCC.

**Conclusion:**

Low-expression ADORA2A-AS1 is correlated with poor survival of HCC patients. ADORA2A-AS1 exerts tumor-suppressive roles in HCC *via* binding HuR and repressing FSCN1/AKT axis.

## Introduction

Liver cancer is one of the most aggressive malignancies, whose mortality nearly matches the incidence, with estimated 905,677 new cases and 830,180 deaths in 2020 globally (1). Hepatocellular carcinoma (HCC) is the major histological subtype of liver cancer (2). Although some advances have been achieved in the therapy of HCC, post-surgical recurrence or metastasis is still common for HCC patients, which leads to poor outcome of HCC (3, 4). Accordingly, it is urgent to elucidate the molecular mechanisms driving HCC development and establish new molecular targets for successful intervention.

Many genetic and epigenetic aberrations are involved in HCC initiation and progression (5, 6). Previous studies about HCC mainly focused on protein targets (7). However, protein-coding sequences only account for less than 2% of the human genome. About 70%~80% of human genome sequences transcribe into transcripts (8). Thus, noncoding transcripts are much more numerous than protein-coding mRNAs (9, 10). Among noncoding transcripts, long noncoding RNA (lncRNA) is the most numerous, which has over 200 nucleotides in length (11, 12). Accumulating evidence has documented that many lncRNAs were aberrantly expressed in various diseases, particular malignancies (13–16). Furthermore, many lncRNAs were documented to play critical roles in various pathophysiological processes including cancers (17–23). These cancer-related lncRNAs exert multiple oncogenic or tumor-suppressive functions, such as cellular proliferation, apoptosis, migration, invasion, drug resistance, and metastasis (24–26).

As a class of crucial regulatory RNAs, lncRNAs regulate the expression of target genes at transcriptional, posttranscriptional, translational, or posttranslational levels (27, 28). For example, lncRNAs bind and recruit epigenetic modification enzymes to target genes to regulate transcription of target genes (29, 30). LncRNAs directly bind mRNAs and regulate the stability and/or translation of the interacted mRNAs (31). Some lncRNAs were found to directly bind microRNAs and abrogate the repressive roles of microRNAs on mRNA stability and/or translation (32, 33). Other lncRNAs were also reported to bind proteins and change posttranslational modification of the interacted proteins, such as ubiquitination and phosphorylation (34, 35). These various roles and mechanisms of lncRNAs and the numerous number of lncRNAs suggested that further mining cancer-related lncRNAs may provide potential candidates for HCC targeting.

Here, *via* analyzing the RNA-sequencing (RNA-seq) data of HCC tissues from The Cancer Genome Atlas (TCGA) project, we identified a novel HCC-related lncRNA ADORA2A-AS1. We further investigated clinical significances, biological roles, and mechanisms of action of ADORA2A-AS1 in HCC in detail.

## Materials and Methods

### Public Datasets and Bioinformatics Analyses

RNA-seq data of HCC tissues and clinicopathological characteristics of these HCCs were retrieved from TCGA project using R2 (https://hgserver1.amc.nl/cgi-bin/r2/main.cgi) and OncoLnc (http://www.oncolnc.org/). Two *in silico* tools Coding Potential Assessment Tool (CPAT) (http://lilab.research.bcm.edu/) and TestCode (https://www.bioinformatics.org/sms/testcode.html) were used to calculate coding potential of ADORA2A-AS1 (36). The proteins bound to ADORA2A-AS1 were predicted using three *in silico* tools, RBPmap (mapping binding sites of RNA binding proteins) (http://rbpmap.technion.ac.il/), ATtRACT (a database of RNA binding proteins and associated motifs) (https://attract.cnic.es/#), and RNA-Protein Interaction Prediction (RPISeq) (http://pridb.gdcb.iastate.edu/RPISeq/index.html) (37, 38).

### Clinical Subjects

Surgically resected HCC tissues (n = 76) were obtained from the Affiliated Hospital of Youjiang Medical University for Nationalities (Baise, China). Informed consents were acquired from all HCC patients. Clinicopathological characteristics of these 76 HCC cases were presented in **Table 1**. The Ethics Committee of Affiliated Hospital of Youjiang Medical University for Nationalities approved this study.

**Table 1:** Correlation between ADORA2A-AS1 expression and clinicopathological characteristics in 76 HCC cases.

Feature	ADORA2A-AS1		χ^2^	p-value
	Low	High		
Age			0.864	0.353
>50	24	20		
≤50	14	18		
Gender			0.461	0.497
Male	34	32		
Female	4	6		
HBs antigen			0.291	0.589
Positive	28	30		
Negative	10	8		
Liver cirrhosis			0.234	0.629
With	14	12		
Without	24	26		
Alpha fetoprotein			1.943	0.163
>20	25	19		
≤20	13	19		
Differentiation			1.983	0.159
I–II	12	18		
III–IV	26	20		
Encapsulation			6.703	0.035
Complete	3	10		
Not complete	20	21		
No	15	7		
Microvascular invasion			4.413	0.036
Absent	18	27		
Present	20	11		
BCLC stage			4.653	0.031
0–A	20	29		
B–C	18	9		

p-value was acquired by Pearson chi-square test. BCLC, Barcelona Clinic Liver Cancer; HCC, hepatocellular carcinoma.

### Cell Culture and Treatment

Human HCC cell lines SNU-398, SK-HEP-1, and Hep3B were obtained from American Type Culture Collection (ATCC, Manassas, VA, USA). SNU-398 cells were cultured in RPMI 1640 medium (Invitrogen, Carlsbad, CA, USA) added with 10% fetal bovine serum (FBS, Invitrogen). SK-HEP-1 and Hep3B cells were cultured in Eagle’s minimum essential medium (Invitrogen) added with 10% FBS. All cells were authenticated by short-tandem repeat analyses. All cells were routinely detected as mycoplasma-free. Where indicated, cells were treated with 50 µM α-amanitin (Sigma-Aldrich, St. Louis, MO, USA) or 10 µM LY294002 (Selleck, Houston, TX, USA) for indicated time.

### Determination of Subcellular Localization of ADORA2A-AS1

RNA fluorescence *in situ* hybridization (FISH) was performed to detect subcellular localization of ADORA2A-AS1 in SNU-398 cells as previously described (31). Briefly, the ADORA2A-AS1 probes were designed and synthesized by Advanced Cell Diagnostics (Newark, NJ, USA). RNA FISH was undertaken using RNAscope Fluorescent Multiplex Detection Kit (Advanced Cell Diagnostics) following the provided instruction. FISH results were detected using confocal laser scanning microscopy (Leica, Wetzlar, Germany). Furthermore, subcellular localization of ADORA2A-AS1 was evaluated using cytoplasmic and nuclear RNA isolation, followed by quantitative real-time polymerase chain reaction (qRT-PCR). Cytoplasmic and nuclear RNA isolation was undertaken using the PARIS Kit (Invitrogen). qRT-PCR was undertaken as described below.

### RNA Extraction and qRT-PCR

Total RNA from HCC tissues and cells was extracted using TRIzol Reagent (Invitrogen). First-strand complementary DNA (cDNA) was generated using the M-MLV Reverse Transcriptase (Invitrogen) and random primers. qRT-PCR was undertaken using the TB Green Premix Ex Taq II (TaKaRa, Tokyo, Japan) on StepOnePlus Real-Time PCR System (Applied Biosystems, Foster City, CA, USA). The sequences of gene-specific primers were as follows: 5′-GGGCTTTCCTACATCCATC-3′ (sense) and 5′-CACAGCAAGTCCATTCCTCT-3′ (anti-sense) for ADORA2A-AS1, 5′-ATCAACCGCCCCATCATC-3′ (sense) and 5′-TGCCCACCGTCCAGTATTT-3′ (anti-sense) for FSCN1, 5′-CCGCAGTGACGGAAAGATG-3′ (sense) and 5′-CCTGGGACAGACGGAAGTT-3′ (anti-sense) for MMP2, 5′-TGGGACCCGTGGGAAGAA-3′ (sense) and 5′-GGCACTTTCAGACTGGACCTC-3′ (anti-sense) for BIRC7, 5′-TGGAGAAATAGTAGATGGC-3′ (sense) and 5′-GGTGAGGAAGTAAAAACAG-3′ (anti-sense) for Metastasis Associated Lung Adenocarcinoma Transcript 1 (MALAT1), 5′-ACACGGACAGGATTGACAGA-3′ (sense) and 5′-GGACATCTAAGGGCATCACA-3′ (anti-sense) for 18S rRNA, 5′-GTCGGAGTCAACGGATTTG-3′ (sense) and 5′-TGGGTGGAATCATATTGGAA-3′ (anti-sense) for glyceraldehyde 3-phosphate dehydrogenase (GAPDH). GAPDH was employed as an internal control. Relative expression levels of transcripts were calculated using the comparative Ct method.

### Vectors and Lentivirus Productions

ADORA2A-AS1 full-length sequences were PCR-amplified with the primers 5′-GGGGTACCTTCGCCTCTCGGTTAGC-3′ (sense) and 5′-CGGGATCCAAAGTTTTAAAACATTCCTGTTAAC-3′ (anti-sense). Next, the PCR products were cloned into the Kpn I and BamH I sites of pcDNA3.1(+) vector (Invitrogen) to construct ADORA2A-AS1 overexpression vector pcDNA3.1-ADORA2A-AS1. The PCR products were also cloned into the Kpn I and BamH I sites of pSPT19 vector (Roche, Basel, Switzerland) to construct ADORA2A-AS1 *in vitro* transcription vector pSPT19-ADORA2A-AS1. Two pairs of cDNA oligonucleotides targeting ADORA2A-AS1 were synthesized and cloned into the shRNA lentivirus expressing plasmid pLV3/H1/GFP&Puro (GenePharma, Shanghai, China). ADORA2A-AS1-specific shRNA lentivirus was produced by GenePharma. Scrambled non-targeting shRNA lentivirus was used as negative control (NC). The sequences of shRNA oligonucleotide were as follows: 5′-GATCCGGATAGAGATCTCAAAGAAGTTTCAAGAGAACTTCTTTGAGATCTCTATCCTTTTTTG-3′ (sense) and 5′-AATTCAAAAAAGGATAGAGATCTCAAAGAAGTTCTCTTGAAACTTCTTTGAGATCTCTATCCG-3′ (anti-sense) for shRNA-ADORA2A-AS1-1, 5′-GATCCGCCCATTGCTTCCAAGCAACATTCAAGAGATGTTGCTTGGAAGCAATGGGCTTTTTTG-3′ (sense) and 5′-AATTCAAAAAAGCCCATTGCTTCCAAGCAACATCTCTTGAATGTTGCTTGGAAGCAATGGGCG-3′ (anti-sense) for shRNA-ADORA2A-AS1-2, 5′-GATCCGTTCTCCGAACGTGTCACGTTTCAAGAGAACGTGACACGTTCGGAGAACTTTTTTG-3′ (sense) and 5′-AATTCAAAAAAGTTCTCCGAACGTGTCACGTTCTCTTGAAACGTGACACGTTCGGAGAACG-3′ (anti-sense) for shRNA-NC.

### Stable Cell Line Constructions

To generate HCC cells with ADORA2A-AS1 stable overexpression, ADORA2A-AS1 overexpression vector pcDNA3.1-ADORA2A-AS1was transfected into SNU-398 and SK-HEP-1 cells using Lipofectamine 3000 (Invitrogen). Empty vector pcDNA3.1 was concurrently transfected and used as negative control. Forty-eight hours after transfection, 800 µg/ml neomycin was added. Individual colonies were picked and expanded. The expression of ADORA2A-AS1 in these colonies was measured using qRT-PCR. We chose one colony with successful ADORA2A-AS1 overexpression for each cell line. To generate HCC cells with ADORA2A-AS1 stable silencing, SUN-398 and Hep3B cells were infected with ADORA2A-AS1-specific shRNA lentivirus. Ninety-six hours after infection, 2 µg/ml puromycin was added. Individual colonies were picked and expanded. The expression of ADORA2A-AS1 in these colonies was measured using qRT-PCR. We chose one colony with successful ADORA2A-AS1 downregulation for each shRNA lentivirus and each cell line. To generate HCC cells with ADORA2A-AS1 and FSCN1 overexpression, ADORA2A-AS1-overexpressing SUN-398 cells were infected with FSCN1 overexpression lentivirus (GenePharma). Ninety-six hours after infection, 2 µg/ml puromycin was added. Individual colonies were picked and expanded. The expression of FSCN1 in these colonies was measured using Western blot.

### Examination of Cell Proliferation, Apoptosis, Migration, and Invasion

Cell proliferation was measured using Cell Counting Kit-8 (CCK-8) and 5-ethynyl-2’-deoxyuridine (EdU) incorporation assays as previously described (39). Briefly, for CCK-8 assay, HCC cells (2,000 per well) were plated into 96-well plate. After culture for 4 days, cell proliferation was measured using CCK-8 reagents (Dojindo, Kumamoto, Japan) following the provided protocol. EdU incorporation assays were performed using the Cell-Light EdU Apollo567 *In Vitro* Kit (RiboBio, Guangzhou, China) following the provided protocol. Cell apoptosis was measured using the Caspase-3 Activity Assay Kit (Cell Signaling Technology, Danvers, MA, USA) following the provided protocol. Cell migration was measured using transwell migration assay as we previously described (40). Cell invasion was measured using transwell invasion assay as we previously described (40).

### Mouse Xenograft Models

Five-week-old male BALB/c nude mice were purchased from Shanghai SLAC Laboratory Animal Co. and maintained in specific pathogen-free conditions. SNU-398 cells with ADORA2A-AS1 overexpression or control were subcutaneously injected into the back flank of nude mice (5 × 10^6^ cells per site). Subcutaneous xenograft volumes were measured every week following the formula volume = 0.5 × length × width^2^. At the 28th day after injection, subcutaneous xenografts were resected, weighed, and subjected to immunohistochemistry (IHC) staining using primary antibodies against Proliferating Cell Nuclear Antigen (PCNA) (#13110, 1:4,000; Cell Signaling Technology), Ki67 (#9027, 1:400; Cell Signaling Technology), or cleaved caspase-3 (#9664, 1:1,000; Cell Signaling Technology). The subcutaneous xenografts were also subjected to terminal deoxynucleotidyl transferase (TdT)-mediated dUTP nick end labeling (TUNEL) assay using the TUNEL Cell Apoptosis Detection Kit (Beyotime, Shanghai, China). SNU-398 cells with ADORA2A-AS1 overexpression or control were also intrasplenically injected into nude mice (2 × 10^6^ cells per mouse). At the 35th day after injection, the livers were resected and subjected to hematoxylin and eosin (H&E) staining to count the diameter and number of metastatic nodules. All animal experiments were approved by the Animal Ethics Committee of Affiliated Hospital of Youjiang Medical University for Nationalities.

### RNA Pull-Down

ADORA2A-AS1 was *in vitro* transcribed from pSPT19-ADORA2A-AS1 using the MEGAscript^®^ Kit (Invitrogen) and Sp6 RNA polymerase (Invitrogen). ADORA2A-AS1 transcript was labeled using the Pierce™ RNA 3′ End Desthiobiotinylation Kit (Thermo Scientific, Waltham, MA, USA). RNA pull-down assays were performed in SNU-398 cells using ADORA2A-AS1 end-labeled with desthiobiotin and the Pierce™ Magnetic RNA-Protein Pull-Down Kit (Thermo Scientific) following the provided protocol. The protein present in the pull-down material was detected by Western blot.

### Western Blot

Proteins extracted from indicated HCC cells or RNA pull-down material were subjected to sodium dodecyl sulfate–polyacrylamide gel electrophoresis and transferred to polyvinylidene fluoride membrane. After blocking, the membranes were incubated with primary antibodies against HuR (Hu Antigen R) (#03-102, 1:500; Millipore, Burlington, MA, USA), FSCN1 (#54545, 1:1,000; Cell Signaling Technology), phosphor-AKT (66444-1-Ig, 1:2,000; Proteintech, Chicago, IL, USA), AKT (10176-2-AP, 1:1,000; Proteintech), MMP2 (#40994, 1:1,000; Cell Signaling Technology), BIRC7 (#5471, 1:1,000; Cell Signaling Technology), or GAPDH (ab8245, 1:5,000; Abcam) overnight at 4°C. The secondary antibodies used were IRDye 680RD Goat anti-Mouse IgG and IRDye 800CW Goat anti-Rabbit IgG (Li-Cor, Lincoln, NE, USA).

### RNA Immunoprecipitation

RNA immunoprecipitation (RIP) assays were undertaken in indicated HCC cells using the Magna RIP RNA-Binding Protein Immunoprecipitation Kit (Millipore) and a primary antibody against HuR (#03-102, 5 µg per reaction; Millipore) following the provided instruction. The precipitated RNAs were measured by qRT-PCR.

### Statistical Analysis

All statistical analyses were performed using the GraphPad Prism 6.0 Software. Log-rank test, Pearson chi-square test, Student’s t-test, one-way ANOVA followed by Dunnett’s multiple comparisons test, and Pearson correlation analysis were used as indicated in the figure and table legends. p < 0.05 was considered statistically significant.

## Results

### Low Expression of ADORA2A-AS1 Was Associated With Advanced Stages and Poor Prognosis in Hepatocellular Carcinoma

**Figure 1:**
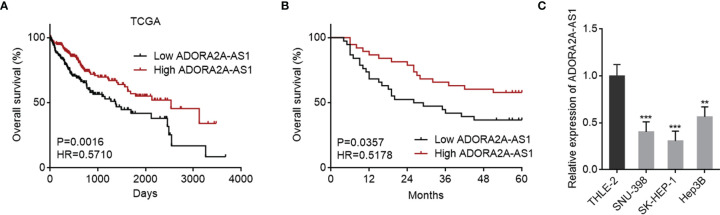
The correlation between ADORA2A-AS1 expression level and outcomes in hepatocellular carcinoma (HCC). **(A)** The correlation between ADORA2A-AS1 expression level and overall survival in HCC analyzed using the RNA sequencing (RNA-seq) data from The Cancer Genome Atlas (TCGA) project. n = 361, p = 0.0016, hazard ratio (HR) = 0.5710 by log-rank test. Median ADORA2A-AS1 expression level was used as cutoff. **(B)** Kaplan–Meier survival analysis of the correlation between ADORA2A-AS1 expression level and overall survival in our HCC cohort. n = 76, p = 0.0357, HR = 0.5178 by log-rank test. Median ADORA2A-AS1 expression level was used as cutoff. **(C)** ADORA2A-AS1 expression levels in immortalized human liver cell THLE-2 and human HCC cells SNU-398, SK-HEP-1, and Hep3B were measured by qRT-PCR. Results are presented as mean ± SD based on three independent experiments. **p < 0.01, ***p < 0.001 by one-way ANOVA followed by Dunnett’s multiple comparisons test.

To explore clinical significances of ADORA2A-AS1 in HCC, we analyzed the RNA-seq data of HCC tissues from TCGA project. The results revealed that low ADORA2A-AS1 expression was associated with worse overall survival of HCC patients (**Figure 1A**). Furthermore, correlation analyses between ADORA2A-AS1 expression and clinicopathological characteristics revealed that low ADORA2A-AS1 expression was associated with advanced American Joint Committee on Cancer (AJCC) pathologic t stage and pathologic tumor stage (**Table 2**). To further confirm the clinical significances of ADORA2A-AS1 in HCC, we randomly collected 76 HCC samples, measured ADORA2A-AS1 expression in these 76 HCC tissues, and analyzed the correlation between ADORA2A-AS1 expression and clinicopathological characteristics. Kaplan–Meier survival analyses revealed that low ADORA2A-AS1 expression was also associated with worse overall survival in our HCC cohort (**Figure 1B**). In this HCC cohort, low ADORA2A-AS1 expression was found to be correlated with bad encapsulation, microvascular invasion, and advanced Barcelona Clinic Liver Cancer (BCLC) stage (**Table 1**). These findings suggested ADORA2A-AS1 as a potential prognostic biomarker for HCC. Furthermore, the expressions of ADORA2A-AS1 in immortalized human liver cell THLE-2 and human HCC cells SNU-398, SK-HEP-1, and Hep3B were measured, and the results revealed that ADORA2A-AS1 was lowly expressed in HCC cells compared with that in immortalized liver cell (**Figure 1C**).

**Table 2:** Correlation between ADORA2A-AS1 expression and clinicopathological characteristics in HCC according to TCGA dataset.

Feature	ADORA2A-AS1		χ^2^	p-value
	Low	High		
Age			0.152	0.697
>50	144	141		
≤50	36	39		
Gender			0.140	0.709
Male	124	120		
Female	57	60		
Child-Pugh			1.272	0.530
A	101	120		
B	9	12		
C	0	1		
Alpha fetoprotein			0.046	0.830
>20	61	68		
≤20	69	73		
Grade			3.321	0.345
G1	25	28		
G2	81	90		
G3	64	57		
G4	8	3		
AJCC tumor pathologic pt			14.476	0.002
T1	71	105		
T2	54	36		
T3	48	31		
T4	8	5		
AJCC pathologic tumor stage			15.551	0.001
I	65	102		
II	48	35		
III	51	31		
IV	2	1		

p-value was acquired by Pearson chi-square test. AJCC, American Joint Committee on Cancer; HCC, hepatocellular carcinoma; TCGA, The Cancer Genome Atlas.

Two *in silico* tools CPAT (http://lilab.research.bcm.edu/) and TestCode (https://www.bioinformatics.org/sms/testcode.html) both indicated that ADORA2A-AS1 was a noncoding RNA (**Figures S1A, B**). RNA FISH results showed that ADORA2A-AS1 was mainly distributed in the cytoplasm (**Figure S1C**). Subcellular fractionation followed by qRT-PCR results also showed that ADORA2A-AS1 was mainly distributed in the cytoplasm (**Figure S1D**).

### ADORA2A-AS1 Repressed Hepatocellular Carcinoma Cellular Proliferation and Promoted Hepatocellular Carcinoma Cellular Apoptosis

**Figure 2:**
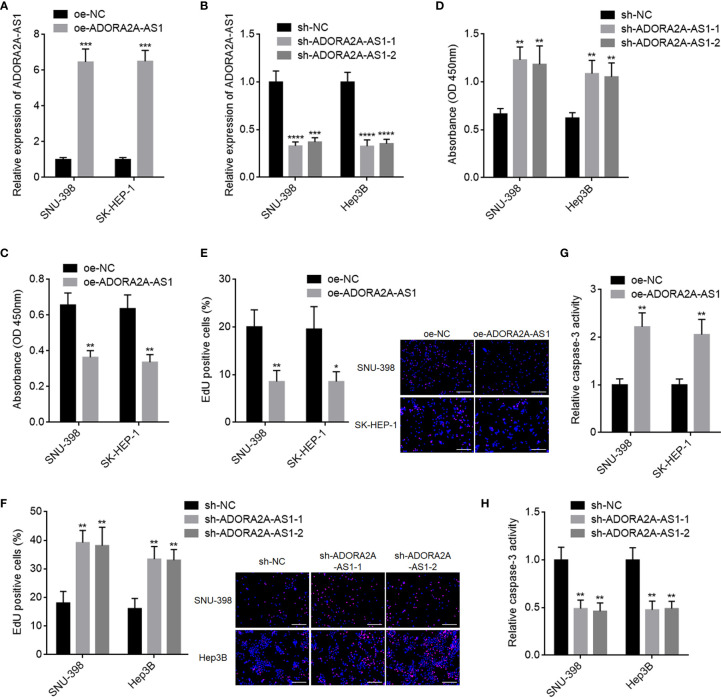
The roles of ADORA2A-AS1 in hepatocellular carcinoma (HCC) cellular proliferation and apoptosis. **(A)** ADORA2A-AS1 expression levels in SNU-398 and SK-HEP-1 cells with ADORA2A-AS1 stable overexpression or control were measured by qRT-PCR. **(B)** ADORA2A-AS1 expression levels in SNU-398 and Hep3B cells with ADORA2A-AS1 stable silencing or control were measured by qRT-PCR. **(C)** Cell proliferation of SNU-398 and SK-HEP-1 cells with ADORA2A-AS1 overexpression or control was evaluated by Cell Counting Kit-8 (CCK-8) assays. **(D)** Cell proliferation of SNU-398 and Hep3B cells with ADORA2A-AS1 stable silencing or control was evaluated by CCK-8 assays. **(E)** Cell proliferation of SNU-398 and SK-HEP-1 cells with ADORA2A-AS1 overexpression or control was evaluated by 5-ethynyl-2’-deoxyuridine (EdU) incorporation assays. Red color indicated EdU incorporated nucleus. Scale bar, 200 µm. **(F)** Cell proliferation of SNU-398 and Hep3B cells with ADORA2A-AS1 stable silencing or control was evaluated by EdU incorporation assays. Red color indicated EdU incorporated nucleus. Scale bar, 200 µm. **(G)** Cell apoptosis of SNU-398 and SK-HEP-1 cells with ADORA2A-AS1 overexpression or control was evaluated by caspase-3 activity assays. **(H)** Cell apoptosis of SNU-398 and Hep3B cells with ADORA2A-AS1 stable silencing or control was evaluated by caspase-3 activity assays. Results are presented as mean ± SD based on three independent experiments. *p < 0.05, **p < 0.01, ***p < 0.001, ****p < 0.0001 by Student’s t-test **(A, C, E, G)** or one-way ANOVA followed by Dunnett’s multiple comparisons test **(B, D, F, H)**.

Due to the correlation between low ADORA2A-AS1 expression and bad encapsulation, microvascular invasion, advanced pathologic t stage, advanced pathologic tumor stage, advanced BCLC stage, and poor overall survival, we next detected the potential roles of ADORA2A-AS1 in HCC. We stably overexpressed ADORA2A-AS1 in SNU-398 and SK-HEP-1 cells *via* stable transfecting of ADORA2A-AS1 overexpression vector (**Figure 2A**). We also stably silenced ADORA2A-AS1 expression in SNU-398 and Hep3B cells *via* stable infecting of ADORA2A-AS1 shRNA lentiviruses (**Figure 2B**). CCK-8 assay results showed that ADORA2A-AS1 overexpression repressed cell proliferation of SNU-398 and SK-HEP-1 cells (**Figure 2C**). Conversely, CCK-8 assay results also showed that ADORA2A-AS1 silencing promoted cell proliferation of SNU-398 and Hep3B cells (**Figure 2D**). EdU incorporation assay results showed that SNU-398 and SK-HEP-1 cells with ADORA2A-AS1 overexpression had less EdU-positive cells compared with their control cells (**Figure 2E**), indicating that ADORA2A-AS1 overexpression repressed cell proliferation of SNU-398 and SK-HEP-1 cells. Conversely, SNU-398 and Hep3B cells with ADORA2A-AS1 silencing had more EdU-positive cells compared with their control cells (**Figure 2F**), indicating that ADORA2A-AS1 silencing promoted cell proliferation of SNU-398 and Hep3B cells. Caspase-3 activity assay results showed that ADORA2A-AS1 overexpression increased caspase-3 activity in SNU-398 and SK-HEP-1 cells (**Figure 2G**), indicating that ADORA2A-AS1 overexpression promoted cell apoptosis of SNU-398 and SK-HEP-1 cells. Conversely, caspase-3 activity assay results also showed that ADORA2A-AS1 silencing decreased caspase-3 activity in SNU-398 and Hep3B cells (**Figure 2H**), indicating that ADORA2A-AS1 silencing repressed cell apoptosis of SNU-398 and Hep3B cells. These findings demonstrated that ADORA2A-AS1 repressed HCC cellular proliferation and promoted HCC cellular apoptosis.

### ADORA2A-AS1 Repressed Hepatocellular Carcinoma Cellular Migration and Invasion

**Figure 3:**
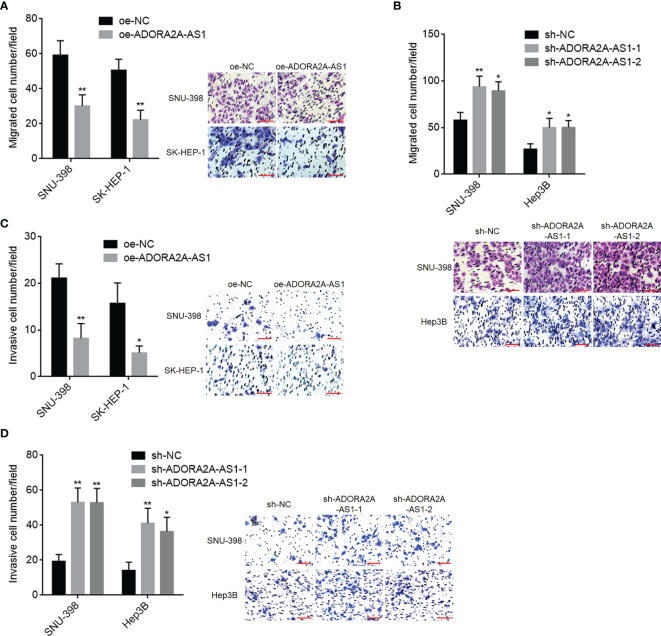
The roles of ADORA2A-AS1 in hepatocellular carcinoma (HCC) cellular migration and invasion. **(A)** Cell migration of SNU-398 and SK-HEP-1 cells with ADORA2A-AS1 overexpression or control was evaluated by transwell migration assays. Scale bar, 100 µm. **(B)** Cell migration of SNU-398 and Hep3B cells with ADORA2A-AS1 stable silencing or control was evaluated by transwell migration assays. Scale bar, 100 µm. **(C)** Cell invasion of SNU-398 and SK-HEP-1 cells with ADORA2A-AS1 overexpression or control was evaluated by transwell invasion assays. Scale bar, 100 µm. **(D)** Cell invasion of SNU-398 and Hep3B cells with ADORA2A-AS1 stable silencing or control was evaluated by transwell invasion assays. Scale bar, 100 µm. Results are presented as mean ± SD based on three independent experiments. *p < 0.05, **p < 0.01 by Student’s t-test **(A, C)** or one-way ANOVA followed by Dunnett’s multiple comparisons test **(B, D)**.

Given that low ADORA2A-AS1 expression was associated with bad encapsulation and microvascular invasion, we next detected the potential roles of ADORA2A-AS1 in HCC cellular migration and invasion. Transwell migration assay results showed that SNU-398 and SK-HEP-1 cells with ADORA2A-AS1 overexpression had less migrated cells compared with their control cells (**Figure 3A**). Conversely, SNU-398 and Hep3B cells with ADORA2A-AS1 silencing had more migrated cells compared with their control cells (**Figure 3B**). Transwell invasion assay results showed that SNU-398 and SK-HEP-1 cells with ADORA2A-AS1 overexpression had less invasive cells compared with their control cells (**Figure 3C**). Conversely, SNU-398 and Hep3B cells with ADORA2A-AS1 silencing had more invasive cells compared with their control cells (**Figure 3D**). These findings demonstrated that ADORA2A-AS1 repressed HCC cellular migration and invasion.

### ADORA2A-AS1 Repressed Hepatocellular Carcinoma Xenograft Growth and Metastasis *In Vivo*

**Figure 4:**
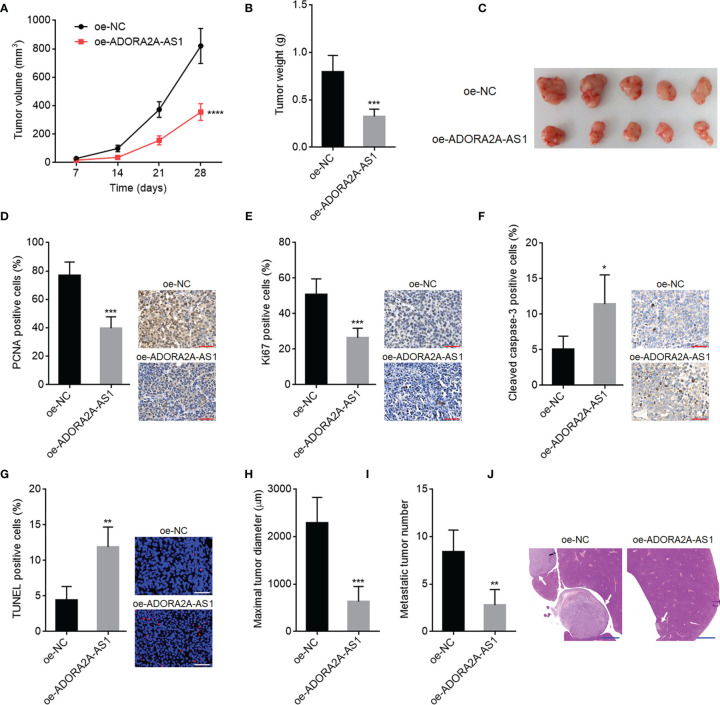
The roles of ADORA2A-AS1 in hepatocellular carcinoma (HCC) xenograft growth and metastasis *in vivo*. **(A)** Subcutaneous xenografts volume over time after injection of SNU-398 cells with ADORA2A-AS1 overexpression or control. **(B)** Subcutaneous xenograft weights at the 28th day after injection of SNU-398 cells with ADORA2A-AS1 overexpression or control. **(C)** The photo of subcutaneous xenografts resected at the 28th day after injection of SNU-398 cells with ADORA2A-AS1 overexpression or control. **(D)** PCNA immunohistochemistry (IHC) staining of subcutaneous xenografts formed by SNU-398 cells with ADORA2A-AS1 overexpression or control. Scale bar, 50 µm. **(E)** Ki67 IHC staining of subcutaneous xenografts formed by SNU-398 cells with ADORA2A-AS1 overexpression or control. Scale bar, 50 µm. **(F)** Cleaved caspase-3 IHC staining of subcutaneous xenografts formed by SNU-398 cells with ADORA2A-AS1 overexpression or control. Scale bar, 50 µm. **(G)** *In vivo* cell apoptosis in subcutaneous xenografts formed by SNU-398 cells with ADORA2A-AS1 overexpression or control was evaluated by terminal deoxynucleotidyl transferase (TdT)-mediated dUTP nick end labeling (TUNEL) assay. Scale bar, 50 µm. **(H–J)** SNU-398 cells with ADORA2A-AS1 overexpression or control were intrasplenically injected into nude mice. At the 35th day after injection, maximal diameter and number of liver metastatic nodules were evaluated by H&E staining. Scale bar, 1,000 µm. Results are presented as mean ± SD based on n = 5 mice in each group. *p < 0.05, **p < 0.01, ***p < 0.001, ****p < 0.0001 by Student’s t-test.

To investigate the roles of ADORA2A-AS1 in mouse xenograft model *in vivo*, SNU-398 cells with ADORA2A-AS1 stable overexpression or control were subcutaneously injected into nude mice. Subcutaneous xenograft growth curve showed that ADORA2A-AS1 overexpression slowed xenograft growth rate (**Figure 4A**). At the 28th day after injection, subcutaneous xenografts were resected and weighed. Obviously, the xenografts formed by ADORA2A-AS1-overexpressed SNU-398 cells were lighter and smaller than the xenografts formed by control SNU-398 cells (**Figures 4B, C** **)**. The xenografts were further subjected to PCNA and Ki67 IHC staining to evaluate cell proliferation *in vivo*. Proliferation markers PCNA and Ki67 IHC staining both showed that ADORA2A-AS1 overexpression repressed cell proliferation *in vivo* (**Figures 4D, E** **)**. Furthermore, cleaved caspase-3 IHC staining of the xenografts showed that ADORA2A-AS1 overexpression promoted cell apoptosis *in vivo* (**Figure 4F**). TUNEL assay results further confirmed that ADORA2A-AS1 overexpression promoted cell apoptosis *in vivo* (**Figure 4G**). To investigate the roles of ADORA2A-AS1 in HCC metastasis, SNU-398 cells with ADORA2A-AS1 stable overexpression or control were intrasplenically injected into nude mice. At the 35th day after injection, liver metastases were evaluated by H&E staining. As shown in **Figures 4H–J**, ADORA2A-AS1-overexpressed SNU-398 cells formed significantly smaller and less liver metastatic nodules. These findings demonstrated that ADORA2A-AS1 repressed HCC xenograft growth and metastasis *in vivo*.

### ADORA2A-AS1 Downregulated FSCN1 *via* Binding HuR

**Figure 5:**
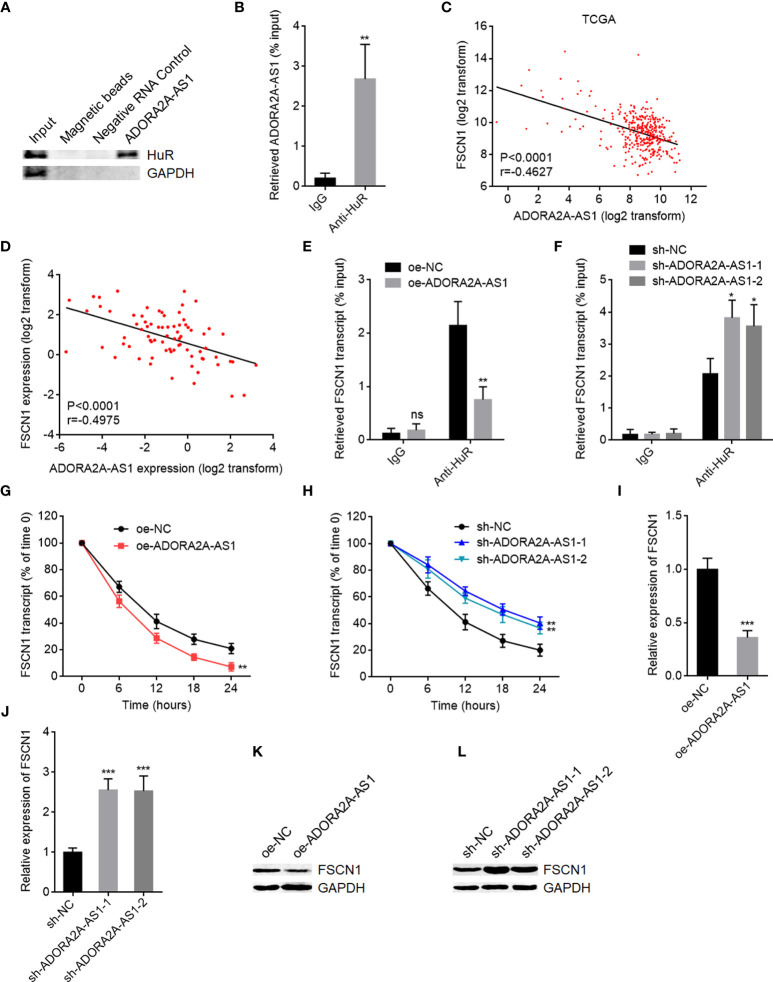
ADORA2A-AS1 upregulated FSCN1 expression *via* binding HuR. **(A)** Proteins were enriched by RNA pull-down assays using ADORA2A-AS1 end-labeled with desthiobiotin, followed by Western blot to detect the binding of HuR to ADORA2A-AS1. **(B)** RIP assays using anti-HuR antibody, followed by qRT-PCR to detect the binding of ADORA2A-AS1 to HuR. **(C)** The correlation between FSCN1 and ADORA2A-AS1 expression in hepatocellular carcinoma (HCC) tissues analyzed using the RNA-seq data from The Cancer Genome Atlas (TCGA) project. n = 360, p < 0.0001, r = -0.4627 by Pearson correlation analysis. **(D)** The correlation between FSCN1 and ADORA2A-AS1 expression in our HCC cohort. n = 76, p < 0.0001, r = -0.4975 by Pearson correlation analysis. **(E)** RIP assays in SNU-398 cells with ADORA2A-AS1 overexpression or control using anti-HuR antibody, followed by qRT-PCR to detect the binding of FSCN1 transcript to HuR. **(F)** RIP assays in SNU-398 cells with ADORA2A-AS1 silencing or control using anti-HuR antibody, followed by qRT-PCR to detect the binding of FSCN1 transcript to HuR. **(G)** FSCN1 transcript stability in SNU-398 cells with ADORA2A-AS1 overexpression or control over time was measured after blocking new RNA transcription using 50 µM α-amanitin. Here, 18S rRNA was used as endogenous control, whose transcription was not repressed by α-amanitin. **(H)** FSCN1 transcript stability in SNU-398 cells with ADORA2A-AS1 silencing or control over time was measured after blocking new RNA transcription using 50 µM α-amanitin. Here, 18S rRNA was used as endogenous control. **(I)** FSCN1 transcript levels in SNU-398 cells with ADORA2A-AS1 overexpression or control were measured by qRT-PCR. **(J)** FSCN1 transcript levels in SNU-398 cells with ADORA2A-AS1 silencing or control were measured by qRT-PCR. **(K)** FSCN1 protein levels in SNU-398 cells with ADORA2A-AS1 overexpression or control were measured by Western blot. **(L)** FSCN1 protein levels in SNU-398 cells with ADORA2A-AS1 silencing or control were measured by Western blot. Results are presented as mean ± SD based on three independent experiments. *p < 0.05, **p < 0.01, ***p < 0.001, ns, not significant, by Student’s t-test **(B, E, G, I)** or one-way ANOVA followed by Dunnett’s multiple comparisons test **(F, H, J)**.

The most common mechanism of action of lncRNAs is to bind proteins and regulate the function of the interacted proteins (20). To investigate whether ADORA2A-AS1 also binds protein and which proteins are bound to ADORA2A-AS1, two *in silico* tools RBPmap (mapping binding sites of RNA binding proteins) (http://rbpmap.technion.ac.il/) and ATtRACT (a database of RNA binding proteins and associated motifs) (https://attract.cnic.es/#) were used to predict the proteins bound to ADORA2A-AS1. Intriguingly, RNA-binding protein HuR was predicted to bind ADORA2A-AS1 by both tools (**Tables S1**, **S2**). The binding of ADORA2A-AS1 to HuR was further predicted by another *in silico* tool RPISeq (RNA-Protein Interaction Prediction) (http://pridb.gdcb.iastate.edu/RPISeq/index.html) with a score of 0.8. RNA pull-down assays using ADORA2A-AS1 end-labeled with desthiobiotin showed that HuR was specifically enriched in ADORA2A-AS1 group (**Figure 5A**). RIP assays using anti-HuR antibody showed that ADORA2A-AS1 was specifically enriched in anti-HuR antibody group (**Figure 5B**). These results demonstrated that ADORA2A-AS1 specifically bound to HuR. HuR is reported to bind and stabilize several mRNAs, including FSCN1, VEGFC, MAT2A, MDM2, CCND1, CASP3, USP7, FAS, PDGFA, CTSS, GATA3, and EGFR (41–46). The RNA-seq data of HCC tissues from TCGA project showed that among all these HuR targets, FSCN1 has the most significant correlation with ADORA2A-AS1 with r value of -0.4627 (**Figure 5C** and **Figure S2**). The significantly negative correlation between ADORA2A-AS1 and FSCN1 expression levels in HCC tissues was also found in our HCC cohort (**Figure 5D**). Therefore, we further explore the potential effects of ADORA2A-AS1 on FSCN1. RIP assays using anti-HuR antibody in SNU-398 cells with ADORA2A-AS1 stable overexpression or control showed that HuR bound to FSCN1 transcript and ADORA2A-AS1 overexpression repressed the binding of HuR to FSCN1 transcript (**Figure 5E**). Conversely, RIP assays using anti-HuR antibody in SNU-398 cells with ADORA2A-AS1 stable silencing or control showed that ADORA2A-AS1 silencing promoted the binding of HuR to FSCN1 transcript (**Figure 5F**). These findings suggested that ADORA2A-AS1 repressed the binding of HuR to FSCN1 transcript *via* competitively binding HuR. Given that HuR could bind and stabilize FSCN1 transcript, we next investigated the effects of ADORA2A-AS1 on FSCN1 transcript stability. SNU-398 cells with ADORA2A-AS1 stable overexpression or silencing were treated with α-amanitin to block new RNA transcription. FSCN1 transcript stability over time was measured, and the results showed that ADORA2A-AS1 overexpression shortened the half-life of FSCN1 transcript **(** **Figure 5G**). Conversely, ADORA2A-AS1 silencing elongated the half-life of FSCN1 transcript (**Figure 5H**). As expected, FSCN1 transcript level was decreased in SNU-398 cells with ADORA2A-AS1 overexpression and increased in SNU-398 cells with ADORA2A-AS1 silencing (**Figures 5I, J** **)**. FSCN1 protein level was also decreased in SNU-398 cells with ADORA2A-AS1 overexpression and increased in SNU-398 cells with ADORA2A-AS1 silencing (**Figures 5K, L** **)**. These findings suggested that ADORA2A-AS1 bound to HuR, repressed the binding of HuR to FSCN1 transcript, reduced FSCN1 transcript stability, and downregulated FSCN1 expression.

### ADORA2A-AS1 Repressed PI3K/AKT Pathway

**Figure 6:**
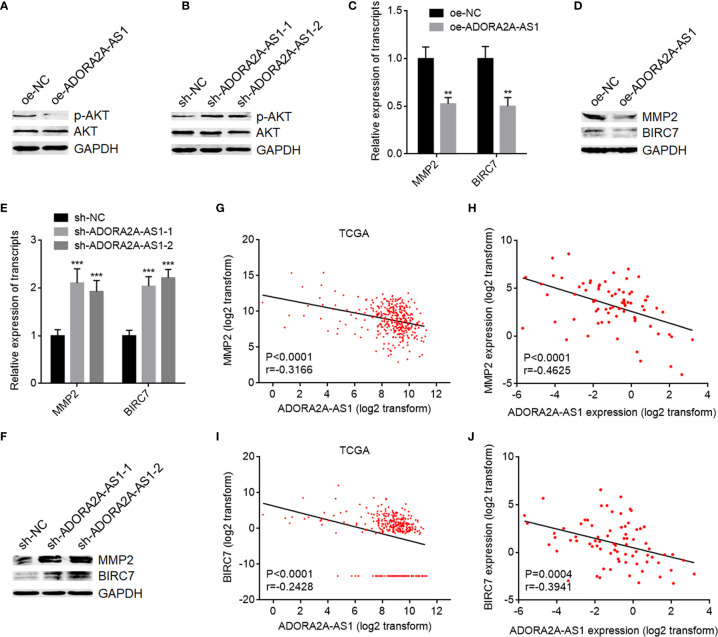
ADORA2A-AS1 repressed AKT pathway. **(A)** AKT phosphorylation levels in SNU-398 cells with ADORA2A-AS1 overexpression or control were measured by Western blot. **(B)** AKT phosphorylation levels in SNU-398 cells with ADORA2A-AS1 silencing or control were measured by Western blot. **(C)** MMP2 and BIRC7 transcript levels in SNU-398 cells with ADORA2A-AS1 overexpression or control were measured by qRT-PCR. **(D)** MMP2 and BIRC7 protein levels in SNU-398 cells with ADORA2A-AS1 overexpression or control were measured by Western blot. **(E)** MMP2 and BIRC7 transcript levels in SNU-398 cells with ADORA2A-AS1 silencing or control were measured by qRT-PCR. **(F)** MMP2 and BIRC7 protein levels in SNU-398 cells with ADORA2A-AS1 silencing or control were measured by Western blot. **(G)** The correlation between MMP2 and ADORA2A-AS1 expression in hepatocellular carcinoma (HCC) tissues analyzed using the RNA-seq data from The Cancer Genome Atlas (TCGA) project. n = 360, p < 0.0001, r = -0.3166 by Pearson correlation analysis. **(H)** The correlation between MMP2 and ADORA2A-AS1 expression in our HCC cohort. n = 76, p < 0.0001, r = -0.4625 by Pearson correlation analysis. **(I)** The correlation between BIRC7 and ADORA2A-AS1 expression in HCC tissues analyzed using the RNA-seq data from TCGA project. n = 360, p < 0.0001, r = -0.2428 by Pearson correlation analysis. **(J)** The correlation between BIRC7 and ADORA2A-AS1 expression in our HCC cohort. n = 76, p = 0.0004, r = -0.3941 by Pearson correlation analysis. For **(C, E)**, results are presented as mean ± SD based on three independent experiments. **p < 0.01, ***p < 0.001 by Student’s t-test **(C)** or one-way ANOVA followed by Dunnett’s multiple comparisons test **(E)**.

FSCN1, also named as Fascin, is an actin-bundling protein (47). High FSCN1 expression has been revealed to be associated with worse outcomes in HCC (48). Increasing evidence showed that FSCN1 is involved in cell proliferation, apoptosis, motility, chemotherapeutic resistance, tumor growth, and metastasis of several malignancies (49, 50). PI3K/AKT pathway was revealed by several reports to be a critical downstream target of FSCN1 (51, 52). Thus, we further investigate the effects of ADORA2A-AS1 on PI3K/AKT pathway. AKT phosphorylation level was decreased in SNU-398 cells with ADORA2A-AS1 overexpression compared with control SNU-398 cells (**Figure 6A**). Conversely, AKT phosphorylation level was increased in SNU-398 cells with ADORA2A-AS1 silencing (**Figure 6B**). MMP2 is a downstream target of AKT activation, which is frequently reported to be involved in cell motility (53). BIRC7, known as Livin, is another critical downstream target of AKT activation, which exerts antiapoptotic roles (51). MMP2 and BIRC7 expression levels were detected in SNU-398 cells with ADORA2A-AS1 overexpression or silencing. As shown in **Figures 6C, D**, mRNA and protein levels of MMP2 and BIRC7 were both decreased in SNU-398 cells with ADORA2A-AS1 overexpression compared with control SNU-398 cells. Conversely, mRNA and protein levels of MMP2 and BIRC7 were both increased in SNU-398 cells with ADORA2A-AS1 silencing (**Figures 6E, F** **)**. The RNA-seq data of HCC tissues from TCGA project showed that MMP2 expression level was negatively correlated with ADORA2A-AS1 expression level (**Figure 6G**). The negative correlation between MMP2 and ADORA2A-AS1 expression levels was also confirmed in our HCC cohort (**Figure 6H**). BIRC7 expression level was also negatively correlated with ADORA2A-AS1 expression level in HCC tissues, analyzed using the RNA-seq data from TCGA project (**Figure 6I**). Our HCC cohort further supported the negative correlation between BIRC7 and ADORA2A-AS1 expression levels (**Figure 6J**). These findings suggested that ADORA2A-AS1 repressed PI3K/AKT pathway *via* downregulating FSCN1.

### FSCN1 Overexpression Attenuated the Tumor-Suppressive Roles of ADORA2A-AS1 in Hepatocellular Carcinoma

**Figure 7:**
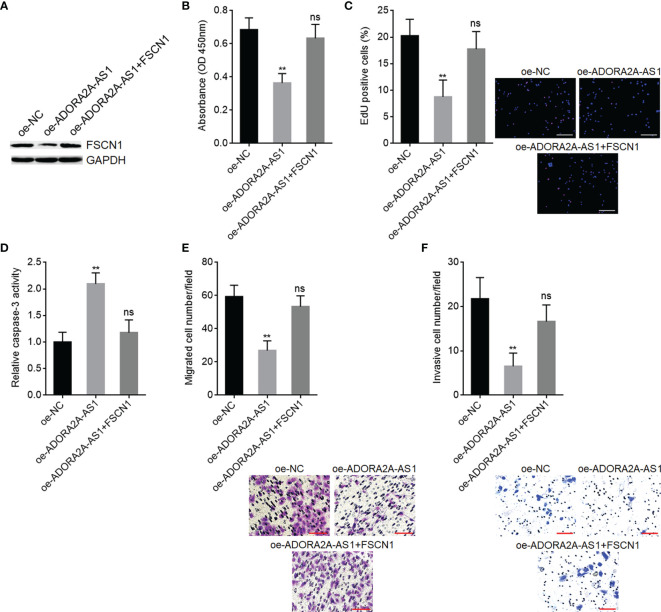
Functional rescue assays using FSCN1 overexpression. **(A)** FSCN1 protein levels in SNU-398 cells with ADORA2A-AS1 and FSCN1 overexpression were measured by Western blot. **(B)** Cell proliferation of SNU-398 cells with ADORA2A-AS1 and FSCN1 overexpression was evaluated by CCK-8 assays. **(C)** Cell proliferation of SNU-398 cells with ADORA2A-AS1 and FSCN1 overexpression was evaluated by 5-ethynyl-2’-deoxyuridine (EdU) incorporation assays. Red color indicated EdU-incorporated nucleus. Scale bar, 200 µm. **(D)** Cell apoptosis of SNU-398 cells with ADORA2A-AS1 and FSCN1 overexpression was evaluated by caspase-3 activity assays. **(E)** Cell migration of SNU-398 cells with ADORA2A-AS1 and FSCN1 overexpression was evaluated by transwell migration assays. Scale bar, 100 µm. **(F)** Cell invasion of SNU-398 cells with ADORA2A-AS1 and FSCN1 overexpression was evaluated by transwell invasion assays. Scale bar, 100 µm. Results are presented as mean ± SD based on three independent experiments. **p < 0.01, ns, not significant, by one-way ANOVA followed by Dunnett’s multiple comparisons test.

To explore whether FSCN1 is the critical mediator of the roles of ADORA2A-AS1 in HCC, we stably overexpressed FSCN1 in SNU-398 cells with ADORA2A-AS1 stable overexpression (**Figure 7A**). CCK-8 assay results showed that FSCN1 overexpression reversed the decreased cell proliferation caused by ADORA2A-AS1 overexpression (**Figure 7B**). EdU incorporation assay results showed that FSCN1 overexpression reversed the decreased EdU-positive cell percentage caused by ADORA2A-AS1 overexpression (**Figure 7C**), indicating that FSCN1 overexpression rescued cell proliferation that was repressed by ADORA2A-AS1. Caspase-3 activity assay results showed that FSCN1 overexpression reversed the increased caspase-3 activity caused by ADORA2A-AS1 overexpression (**Figure 7D**), indicating that FSCN1 overexpression reversed cell apoptosis that was induced by ADORA2A-AS1. Transwell migration assay results showed that FSCN1 overexpression reversed the decreased migrated cell number caused by ADORA2A-AS1 overexpression (**Figure 7E**). Transwell invasion assay results showed that FSCN1 overexpression reversed the decreased invasive cell number caused by ADORA2A-AS1 overexpression (**Figure 7F**). These findings suggested that FSCN1 reversed the roles of ADORA2A-AS1 in repressing cell proliferation, inducing cell apoptosis, and repressing cell migration and invasion.

### Inhibition of PI3K/AKT Pathway Attenuated the Oncogenic Roles of ADORA2A-AS1 Depletion in Hepatocellular Carcinoma

**Figure 8:**
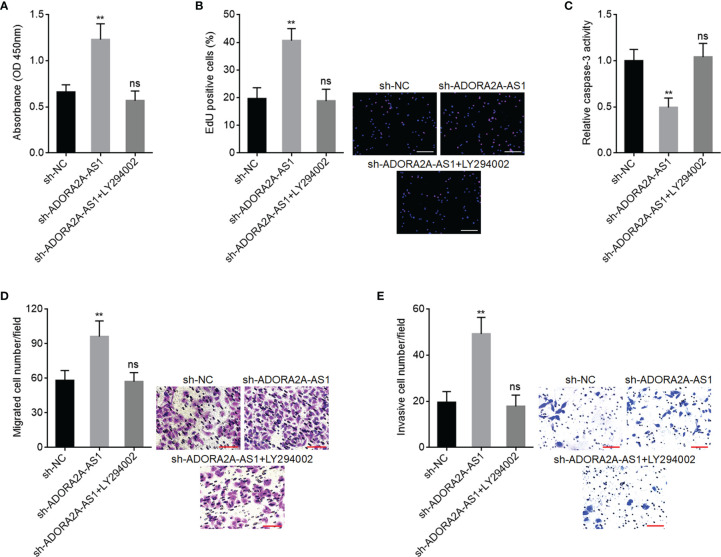
PI3K/AKT pathway inhibitor LY294002 abolished the roles of ADORA2A-AS1 depletion in hepatocellular carcinoma (HCC). **(A)** Cell proliferation of ADORA2A-AS1-silenced SNU-398 cells treated with 10 µM LY294002 was evaluated by Cell Counting Kit-8 (CCK-8) assays. **(B)** Cell proliferation of ADORA2A-AS1-silenced SNU-398 cells treated with 10 µM LY294002 was evaluated by 5-ethynyl-2’-deoxyuridine (EdU) incorporation assays. Red color indicated EdU incorporated nucleus. Scale bar, 200 µm. **(C)** Cell apoptosis of ADORA2A-AS1-silenced SNU-398 cells treated with 10 µM LY294002 was evaluated by caspase-3 activity assays. **(D)** Cell migration of ADORA2A-AS1-silenced SNU-398 cells treated with 10 µM LY294002 was evaluated by transwell migration assays. Scale bar, 100 µm. **(E)** Cell invasion of ADORA2A-AS1-silenced SNU-398 cells treated with 10 µM LY294002 was evaluated by transwell invasion assays. Scale bar, 100 µm. Results are presented as mean ± SD based on three independent experiments. **p < 0.01, ns, not significant, by one-way ANOVA followed by Dunnett’s multiple comparisons test.

To further explore whether PI3K/AKT pathway is also the critical mediator of the roles of ADORA2A-AS1 in HCC, SNU-398 cells with ADORA2A-AS1 stable silencing were treated with PI3K/AKT pathway inhibitor LY294002. CCK-8 assay results showed that LY294002 reversed the increased cell proliferation caused by ADORA2A-AS1 silencing (**Figure 8A**). EdU incorporation assay results showed that LY294002 reversed the increased EdU-positive cell percentage caused by ADORA2A-AS1 silencing (**Figure 8B**), indicating that LY294002 reversed the increased cell proliferation induced by ADORA2A-AS1 silencing. Caspase-3 activity assay results showed that LY294002 reversed the reduced caspase-3 activity caused by ADORA2A-AS1 silencing (**Figure 8C**), indicating that LY294002 reversed the reduced cell apoptosis caused by ADORA2A-AS1 silencing. Transwell migration assay results showed that LY294002 reversed the increased migrated cell number caused by ADORA2A-AS1 silencing (**Figure 8D**). Transwell invasion assay results showed that LY294002 reversed the increased invasive cell number caused by ADORA2A-AS1 silencing (**Figure 8E**). These findings suggested that the oncogenic roles of ADORA2A-AS1 depletion in HCC were abolished by PI3K/AKT pathway inhibition.

## Discussion

In this study, we identified an HCC-related tumor-suppressive lncRNA ADORA2A-AS1. *ADORA2A-AS1* locates at chromosome 22q11.23. ADORA2A-AS1 has limited protein-encoding ability. Low expression of ADORA2A-AS1 in HCC tissues was correlated with advanced stage, bad encapsulation, microvascular invasion, and poor survival of HCC patients. Our findings suggested ADORA2A-AS1 as a potential prognostic biomarker for HCC, which needs further multicentral investigation.

Gain and loss-of functional experiments demonstrated that ADORA2A-AS1 repressed HCC cellular proliferation, induced HCC cellular apoptosis, repressed HCC cellular migration and invasion, and restricted HCC xenograft growth and metastasis *in vivo*. Thus, our findings suggested ADORA2A-AS1 as a tumor-suppressive lncRNA in HCC. Enhancing ADORA2A-AS1 expression represented potential therapeutic strategy against HCC.

Mechanistic investigations identified HuR as a critical interaction partner of ADORA2A-AS1. HuR, also named ELAVL1 (embryonic lethal, abnormal vision Drosophila-like protein 1), is an RNA-binding protein. HuR posttranscriptionally regulates expression of target genes *via* directly binding target mRNAs and further stabilizing target mRNAs (54). Previous reports have shown that high expression of HuR was associated with poor survival in HCC (45). HuR promotes HCC malignant progression *via* regulating several mRNA targets (45).

Combining bioinformatics prediction, RNA pull-down, and RIP assays, we documented the specific physical binding between ADORA2A-AS1 and HuR. The binding of ADORA2A-AS1 to HuR competitively suppressed the binding of HuR to its mRNA targets. Here, we identified FSCN1 as one of the most significantly changed mRNA targets regulated by ADORA2A-AS1. ADORA2A-AS1 competitively suppressed the binding of HuR to FSCN1 transcript, leading to the reduction of FSCN1 transcript stability. Thus, ADORA2A-AS1 downregulated FSCN1 expression. The expression of FSCN1 was significantly negatively correlated with ADORA2A-AS1 in HCC tissues, which was verified in both TCGA project and our own HCC cohort. According to the RNA-seq data of TCGA project, the expression abundance of ADORA2A-AS1 was similar with that of FSCN1 transcript in HCC tissues, supporting the competition between ADORA2A-AS1 and FSCN1 transcript to bind HuR. Potential effects of ADORA2A-AS1 on other HuR targets need further investigation.

Conversely to ADORA2A-AS1, high expression of FSCN1 was correlated with poor outcome of HCC patients (48). FSCN1 was documented to exert oncogenic roles in several malignancies, including HCC (49, 50). For example, FSCN1 was reported to promote HCC cellular proliferation, migration, and invasion, inhibit HCC cellular apoptosis, and increase HCC cellular doxorubicin resistance (55–58). PI3K/AKT pathway is the classically oncogenic pathway, which has been documented to modulate cell proliferation, survival, and motility in various malignancies (59–62). PI3K/AKT pathway was also revealed to be the critical downstream target of FSCN1 (51). Consistently, in this study, we found that ADORA2A-AS1 repressed PI3K/AKT pathway through downregulating FSCN1. MMP2 and BIRC7 are two representative targets of FSCN1/AKT (51, 53). In this study, we further found that ADORA2A-AS1 downregulated MMP2 and BIRC7 *via* repressing FSCN1/AKT axis. The expressions of MMP2 and BIRC7 were both negatively correlated with ADORA2A-AS1 in HCC tissues, verified in both TCGA project and our own HCC cohort. Functional rescue experiments showed that FSCN1/AKT axis was the critical mediator of the roles of ADORA2A-AS1 in HCC. We acknowledge that this study also has some limitations, such as the potential effects of ADORA2A-AS1 on other HuR targets and FSCN1 targets. Nevertheless, FSCN1/AKT axis is one of the critical mediators of the functions of ADORA2A-AS1 in HCC.

In conclusion, ADORA2A-AS1 is identified as a novel HCC-related tumor-suppressive lncRNA, whose low expression is correlated with poor outcome of HCC patients. ADORA2A-AS1 exerts tumor-suppressive roles in HCC *via* competitively binding HuR, decreasing the binding of HuR to FSCN1 transcript, downregulating FSCN1 transcript stability, repressing FSCN1 expression, and repressing AKT pathway. ADORA2A-AS1/HuR/FSCN1/AKT represent potential therapeutic targets for HCC.

## Data Availability Statement

The original contributions presented in the study are included in the article/**Supplementary Material**. Further inquiries can be directed to the corresponding author.

## Ethics Statement

The studies involving human participants were reviewed and approved by the Ethics Committee of Affiliated Hospital of Youjiang Medical University for Nationalities. The patients/participants provided their written informed consent to participate in this study. The animal study was reviewed and approved by the Animal Ethics Committee of Affiliated Hospital of Youjiang Medical University for Nationalities.

## Author Contributions

HW and JP designed the study. JP, YZ, AW, ZQ, CZ, WL, and ZX undertook the experiments. JP, HW, YZ, QT, and JW analyzed the data. JP and HW are the major contributors in writing the manuscript. All authors contributed to the article and approved the submitted version.

## Funding

This work was supported by the Guangxi Natural Science Foundation Project (2019GXNSFBA245023), Guangxi Science and Technology Project (AD17129025), and Baise Scientific Research and Technology Development Programme (BKZ2020-47-23).

## Conflict of Interest

The authors declare that the research was conducted in the absence of any commercial or financial relationships that could be construed as a potential conflict of interest.

## Publisher’s Note

All claims expressed in this article are solely those of the authors and do not necessarily represent those of their affiliated organizations, or those of the publisher, the editors and the reviewers. Any product that may be evaluated in this article, or claim that may be made by its manufacturer, is not guaranteed or endorsed by the publisher.
