# Supplementary material for: ADORA2A-AS1 Restricts Hepatocellular Carcinoma Progression via Binding HuR and Repressing FSCN1/AKT Axis
Source: Front Oncol. 2021 Oct 18;11:754835. doi: 10.3389/fonc.2021.754835 (PMC8558402; doi:10.3389/fonc.2021.754835)
Supplement: Supplementary file 1 [file DataSheet_1.docx]

Supplementary Material

# Supplementary Figures and Tables

## Supplementary Figures


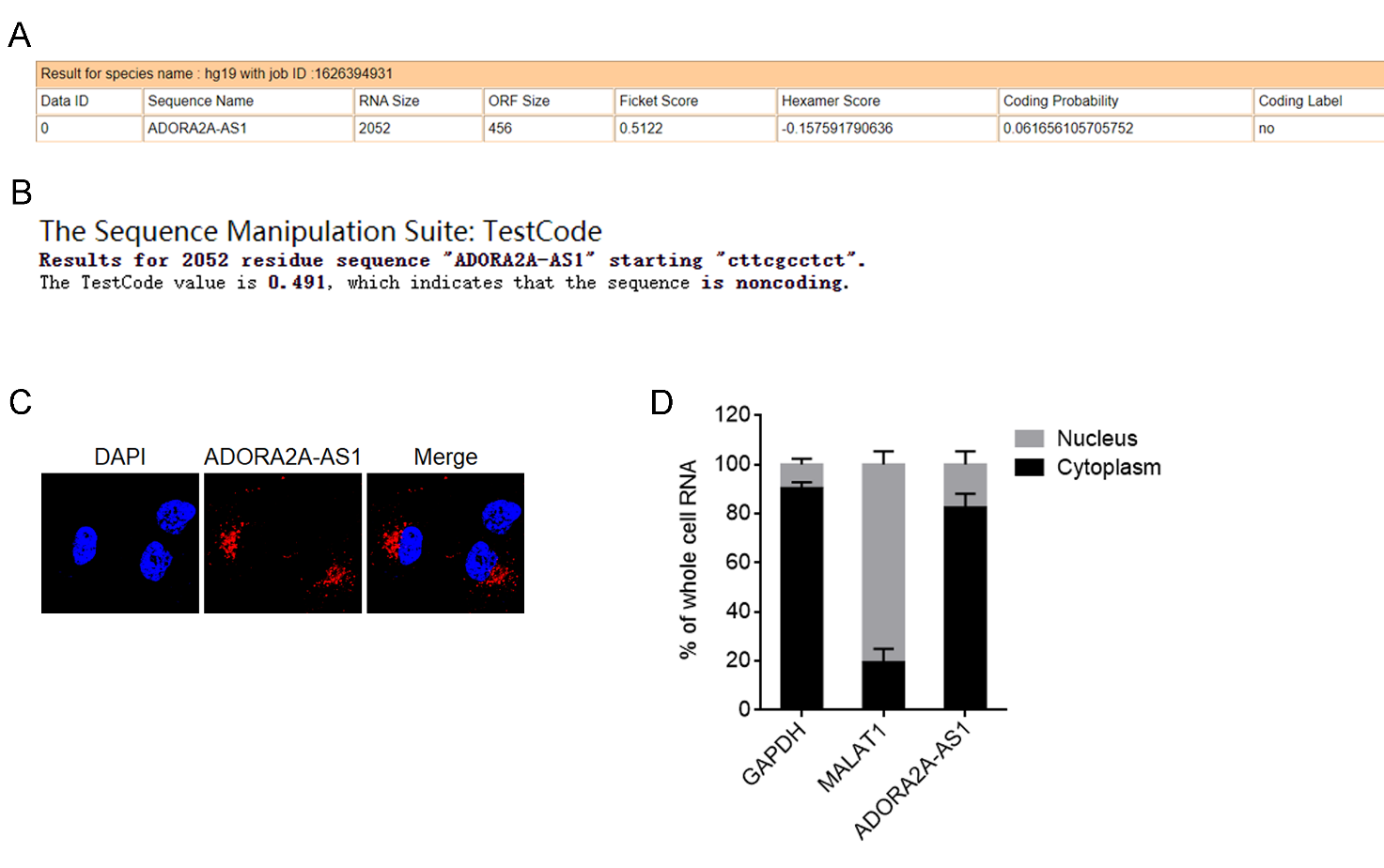


**Supplementary Figure 1.** The characteristics of ADORA2A-AS1. **(A)** The coding potential of ADORA2A-AS1 was calculated using Coding Potential Assessment Tool (CPAT). **(B)** The coding potential of ADORA2A-AS1 was calculated using TestCode. **(C)** Subcellular distribution of ADORA2A-AS1 in SNU-398 cells was detected by RNA FISH. **(D)** Subcellular distribution of ADORA2A-AS1 in SNU-398 cells was detected by subcellular fractionation, followed by qRT-PCR. Results are presented as mean ± SD based on three independent experiments.


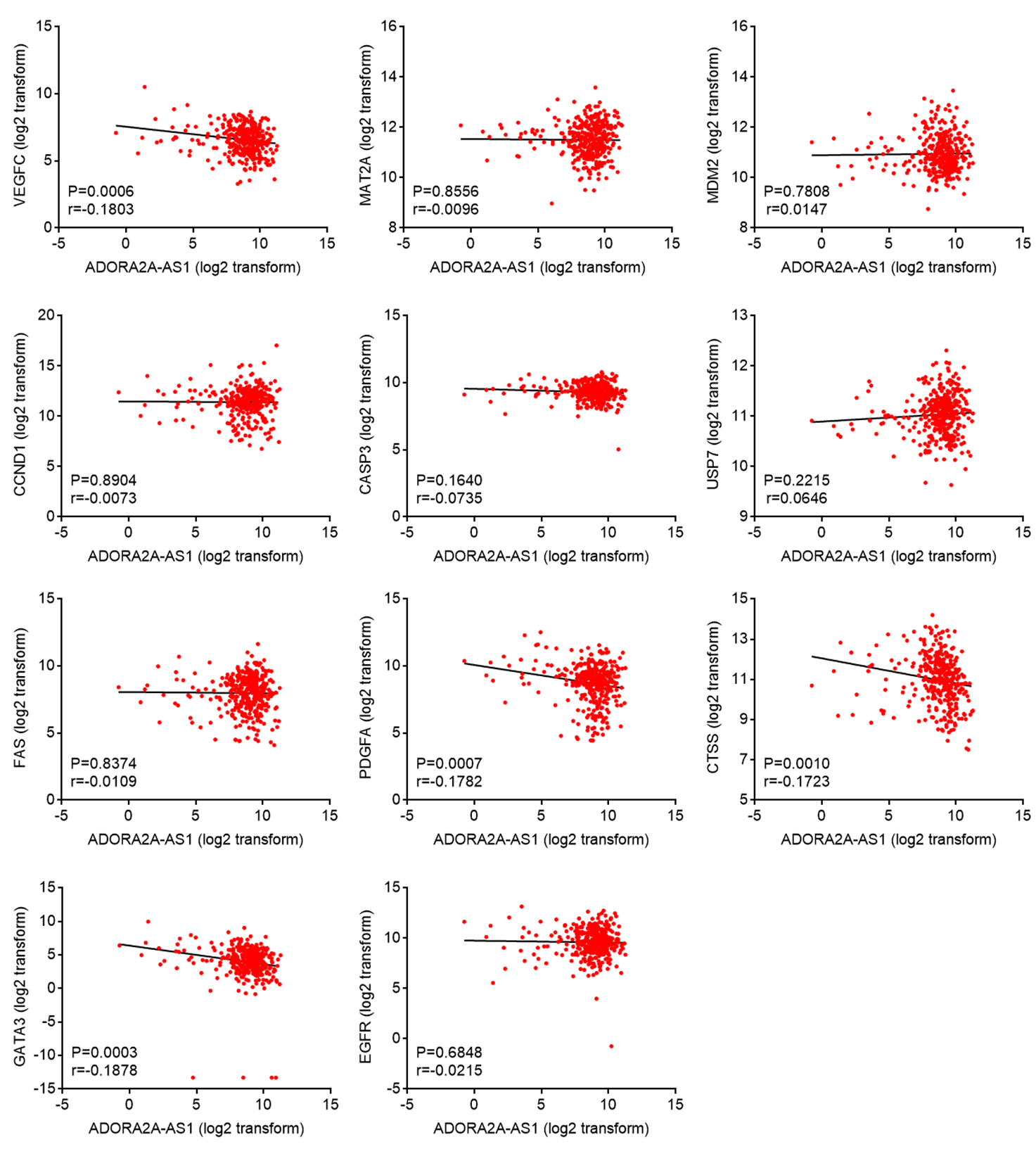


**Supplementary Figure 2.** The correlation between HuR targets and ADORA2A-AS1 expression in HCC tissues analyzed using the RNA-seq data from TCGA project. P values were calculated by Pearson correlation analyses.

## Supplementary Tables

**Supplementary Table 1.** The binding of HuR to ADORA2A-AS1 Predicted by RBPmap.

| Sequence Position | Motif | K-mer | Z-score | P-value |
| --- | --- | --- | --- | --- |
| 1272 | uukruuu | uuugauu | 2.121 | 1.70e-02 |
| 1273 | uukruuu | uugauuc | 2.121 | 1.70e-02 |
| 1948 | uukruuu | uuaauuu | 2.143 | 1.61e-02 |
| 1987 | uukruuu | cuggguu | 1.989 | 2.34e-02 |
| 2012 | uukruuu | auguuuu | 2.176 | 1.48e-02 |

**Supplementary Table 2.** The binding of HuR to ADORA2A-AS1 Predicted by ATtRACT.

| Gene Name | Motif | Experiment | PubMed | Domain | Off Set |
| --- | --- | --- | --- | --- | --- |
| ELAVL1 | AUUU | X-ray diffraction | 23519412 | RRM | 1915;393; 983;1951 |
| ELAVL1 | AUUUA | UV crosslink, immunoblotting with HeLa nuclear extracts, EMSA with recombinant protein. | 9882309 | RRM | 1915 |
| ELAVL1 | AUUUU | X-ray diffraction | 23519412 | RRM | 1951 |
| ELAVL1 | UUUU | X-ray diffraction | 23519412 | RRM | 2015;1952; 1271 |
